# Translocation of a parthenogenesis gene candidate to an alternate carrier chromosome in apomictic *Brachiaria humidicola*

**DOI:** 10.1186/s12864-018-5392-4

**Published:** 2019-01-14

**Authors:** Margaret Worthington, Masumi Ebina, Naoki Yamanaka, Christopher Heffelfinger, Constanza Quintero, Yeny Patricia Zapata, Juan Guillermo Perez, Michael Selvaraj, Manabu Ishitani, Jorge Duitama, Juan Fernando de la Hoz, Idupulapati Rao, Stephen Dellaporta, Joe Tohme, Jacobo Arango

**Affiliations:** 1 International Center for Tropical Agriculture (CIAT), A.A. 6713, Cali, Colombia; 2 National Agriculture and Food Research Organization (NARO), Institute of Livestock and Grassland Science, Nasushiobara, Tochigi 392-2793 Japan; 3 Japan International Research Center for Agricultural Sciences (JIRCAS), 1-1 Ohwashi, Tsukuba, Ibaraki 305-8686 Japan; 4 Department of Molecular, Cellular, and Developmental Biology, Yale University, New Haven, CT 06520 USA; 5 Present address: Department of Horticulture, University of Arkansas, 306 Plant Sciences Bldg, Fayetteville, AR 72701 USA; 6 Present address: Systems and Computing Engineering Department, Universidad de los Andes, Bogotá, Colombia; 7 Present address: Bioinformatics Interdepartmental Ph.D. Program, University of California, Los Angeles, Los Angeles, CA 90095 USA; 8 Present address: Plant Polymer Research Unit (PPL), National Center for Agricultural Utilization Research (NCAUR), Agricultural Research Service, United States Department of Agriculture (ARS-USDA), 1815 N. University St., Peoria, IL 61604 USA

**Keywords:** Apomixis, Apospory, Parthenogenesis, BABY BOOM, Aneuploidy, Segmental allopolyploidy, Genotyping-by-sequencing, Molecular karyotyping, *Urochloa*, *Brachiaria*

## Abstract

**Background:**

The apomictic reproductive mode of *Brachiaria* (syn. *Urochloa*) forage species allows breeders to faithfully propagate heterozygous genotypes through seed over multiple generations. In *Brachiaria*, reproductive mode segregates as single dominant locus, the apospory-specific genomic region (ASGR). The AGSR has been mapped to an area of reduced recombination on *Brachiaria decumbens* chromosome 5. A primer pair designed within *ASGR-BABY BOOM-like* (*BBML*), the candidate gene for the parthenogenesis component of apomixis in *Pennisetum squamulatum,* was diagnostic for reproductive mode in the closely related species *B. ruziziensis*, *B. brizantha*, and *B. decumbens*. In this study, we used a mapping population of the distantly related commercial species *B. humidicola* to map the ASGR and test for conservation of *ASGR-BBML* sequences across *Brachiaria* species.

**Results:**

Dense genetic maps were constructed for the maternal and paternal genomes of a hexaploid (2n = 6x = 36) *B. humidicola* F_1_ mapping population (*n* = 102) using genotyping-by-sequencing, simple sequence repeat, amplified fragment length polymorphism, and transcriptome derived single nucleotide polymorphism markers. Comparative genomics with *Setaria italica* provided confirmation for x = 6 as the base chromosome number of *B. humidicola*. High resolution molecular karyotyping indicated that the six homologous chromosomes of the sexual female parent paired at random, whereas preferential pairing of subgenomes was observed in the apomictic male parent. Furthermore, evidence for compensated aneuploidy was found in the apomictic parent, with only five homologous linkage groups identified for chromosome 5 and seven homologous linkage groups of chromosome 6. The ASGR mapped to *B. humidicola* chromosome 1, a region syntenic with chromosomes 1 and 7 of *S. italica*. The *ASGR-BBML* specific PCR product cosegregated with the ASGR in the F_1_ mapping population, despite its location on a different carrier chromosome than *B. decumbens*.

**Conclusions:**

The first dense molecular maps of *B. humidicola* provide strong support for cytogenetic evidence indicating a base chromosome number of six in this species. Furthermore, these results show conservation of the ASGR across the Paniceae in different chromosomal backgrounds and support postulation of the *ASGR-BBML* as candidate genes for the parthenogenesis component of apomixis.

**Electronic supplementary material:**

The online version of this article (10.1186/s12864-018-5392-4) contains supplementary material, which is available to authorized users.

## Background

Several important forage grass genera, including *Brachiaria* (Trin.) Griseb. (syn. *Urochloa* P. Beauv.), *Cenchrus* L./*Pennisetum* Rich., *Panicum* L. (syn. *Megathrysus*), and *Paspalum* L., reproduce via apomixis. Apomixis, asexual reproduction through seed, is a naturally occurring reproductive mode that enables breeders to select and faithfully propagate outstanding heterozygous genotypes without vegetative propagation or hybrid seed production. In apospory, the apomictic pathway found in *Brachiaria* and other Paniceae grass genera, unreduced embryo sacs first develop from an adjacent somatic nucellar cell in a process termed apomeiosis [1]. This unreduced embryo sac then develops into a viable embryo without fertilization through parthenogenesis [2, 3]. Pseudogamous fertilization of the secondary nuclei of apomictic embryo-sacs by viable pollen gametes is required for normal endosperm development to occur [3].

The apomeiosis and parthenogenesis components of apomixis are usually inherited together as a single dominant Mendelian factor denoted as the “apospory-specific genomic region” (ASGR) in Paniceae grasses [1]. The ASGR was mapped to a physically large hemizygous region of reduced recombination in *Pennisetum squamulatum* (L.) R. Br. (syn. *Cenchrus squamulatus*) [4], and *Paspalum notatum* Flüggé and *Paspalum simplex* Morong [5–9]. The *ASGR-BABY BOOM-like* (*ASGR-BBML*) genes have been posited as candidate genes for parthenogenesis in *Cenchrus*/*Pennisetum* [10]. The *ASGR-BBML* have high sequence similarity to the *BABY BOOM* (*BBM*) genes associated with somatic embryogenesis in *Brassica* and *Arabidopsis* [11]*.* Furthermore, apomictic F_1_ RNAi transgenic plants with reduced *PsASGR-BBML* expression had reduced parthenogenic embryo development and transgenic sexual pearl millet plants expressing *P. squamulatum PsASGR-BBML* genes were induced to produce haploid offspring [10].

A recent study in the genus *Brachiaria* provided additional support for the postulation of the *ASGR-BBML* as candidate genes for the apomictic function of parthenogenesis [12]. The *psASGR–BBML*-specific primer pair p779/p780 was developed from sequences in the 4th and 7th exons of *P. squamulatum ASGR-BBM-like2* [13]. This marker was previously linked to the ASGR in F_1_ populations developed with *P. squamulatum* and *C. ciliaris* as apomictic pollen parents and validated in a diversity panel of apomictic and sexual *Pennisetum* and *Cenchrus* species [13]. The p779/p780 amplicon cosegregated with reproductive mode in a large interspecific *B. ruziziensis* (R. Germ. and C. M. Evrard) x *B. decumbens* Stapf. F_1_ mapping population and mapped to a region of reduced recombination on *B. decumbens* chromosome 5 [12]. The *psASGR–BBML*-specific amplicon was also diagnostic for apomixis in a panel of CIAT genebank accessions from the closely related *B. brizantha* (A. Rich.) Stapf, *B. decumbens*, *B. ruziziensis* agamic complex with known reproductive mode [12].

Less is known about the genetic control of apomixis in *B. humidicola* (Rendle) Schweick (koroniviagrass, syn. *Urochloa humidicola* (Rendle) Morrone & Zuloaga). Like the other economically important *Brachiaria* species (*B. brizantha*, *B. decumbens*, and *B. ruziziensis*), *B. humidicola*, native to East Africa, was introduced to tropical Latin America during the mid-twentieth century [14]. *Brachiaria humidicola* is highly stoloniferous and well adapted to regions with infertile acidic soil, poor drainage, and seasonal waterlogging [15]. *Brachiaria humidicola* is estimated to have diverged from the other commercial *Brachiaria* species in the *B. brizantha*/*B. decumbens*/*B. ruziziensis* agamic complex about 9.46 mya [16]. The distant relationship between *B. humidicola* and the other commercial *Brachiaria* species is supported by marked differences in inflorescence morphology [14] and a recent phylogenetic study that assessed a large interspecific collection of 261 *Brachiaria* genotypes with STRUCTURE, neighbor joining (NJ), unweighted pair group method with arithmetic mean (UPGMA), and multiple correspondence analyses [17]. No successful crosses have ever been documented between *B. humidicola* and any members of the *B. brizantha/B. decumbens*/*B. ruziziensis* agamic complex, suggesting that the species are sexually incompatible.

While most *Brachiaria* species are reported to have a base chromosome number of x = 9 [12, 18–22], cytogenetic evidence suggests that the base chromosome number of *B. humidicola* and its close relative *B. dictyoneura* is x = 6. Specific evidence for x = 6 as the base chromosome number for these species included the presence of hexavalents in accessions with 2n = 36 and 2n = 42 chromosomes and octa- and nonavalents in accessions with 2n = 54 chromosomes [23–25]. An allopolyploid origin for *B. humidicola* (AAAABB) was suggested based on meiotic analyses and marker segregation behavior in an simple sequence repeat (SSR)-based linkage map developed from a hexaploid (2n = 6x = 36) population derived from a cross of the sexual polyploid *B. humidicola* accession (CIAT 26146) and the apomictic cultivar ‘Tupi’ [26].

*Brachiaria humidicola* exists primarily as a polyploid apomict in nature. Controlled crosses of *B. humidicola* were first made possible by the discovery of a natural sexual polyploid accession held in the CIAT and EMBRAPA germplasm collections [27]. This sexual polyploid accession, CIAT 26146 (H031 EMBRAPA Beef Cattle) was determined to have 36 chromosomes and used as a female parent in crosses with eighteen apomictic *B. humidicola* accessions determined to have hexaploid DNA content by flow cytometry [28]. The progeny from these crosses formed the basis of the CIAT *B. humidicola* breeding program, which is focused on the development of apomictic cultivars with improved forage quality, productivity, and seed yield.

Only apomictic genotypes can be released as uniform, true-breeding cultivars, while only sexually reproducing genotypes can be used as female parents to develop segregating populations [29]. Because apospory segregates as a single dominant Mendelian factor, each cross between a sexually reproducing female parent and an apomictic pollen donor is expected to produce progeny segregating for reproductive mode on a 1:1 basis. Phenotypic evaluation of reproductive mode in large segregating populations through progeny tests or embryo sac analysis is expensive and time-consuming. Thus, the development of a diagnostic marker test for reproductive mode could shorten breeding cycles and reduce costs [30]. CIAT breeders have used ‘N14’, a sequence characterized amplified region (SCAR) marker in linkage with the ASGR for routine evaluation of reproductive mode in seedlings from the interspecific *B. brizantha/B. decumbens*/*B. ruziziensis* breeding program [30, 31]. Random amplified polymorphic DNA (RAPD) primers producing a band linked to reproductive mode in a *B. humidicola* mapping population have also been reported [32]. Unfortunately, neither ‘N14’ nor the linked RAPD marker produced polymorphic bands linked to the ASGR in CIAT *B. humidicola* breeding populations. Fifty-two *B. humidicola* accessions held in the CIAT genetic resources collection were genotyped with the *psASGR–BBML*-specific primer pair p779/p780, and 950 bp amplicons were produced by all accessions except CIAT 26146, the only sexual accession [12]. This finding suggests that p779/p780 might be diagnostic in *B. humidicola* as well as the agamic complex species. However, p779/p780 has not yet been tested for linkage with the ASGR in segregating *B. humidicola* populations.

Polyploidy, multisomic inheritance, heterozygosity, and self-incompatibility have slowed progress in *Brachiaria* genomics, but recent advances such as genotyping-by-sequencing (GBS) and bioinformatics pipelines for species lacking reference genomes make the construction of dense maps possible in polyploid apomict species. The primary objective of this study was to develop linkage maps of *B. humidicola* using a hexaploid (2n = 6x = 36) F_1_ mapping population segregating for reproductive mode. These maps were used to assess synteny with the related species foxtail millet (*Setaria italica* (L.) P. Beauv) and evaluate meiotic interactions among homologous and homeologous linkage groups. The paternal linkage map was also used to locate the position of the ASGR and validate whether p779/p780 is diagnostic for reproductive mode in *B. humidicola*.

## Methods

### Materials

A biparental population of 124 F_1_ progeny was generated by crossing the sexual accession CIAT 26146 [EMBRAPA Beef Cattle (EBC) H031] to the apomictic male parent CIAT 16888 (EBC H027). The female and male parents of the cross are natural germplasm accessions collected in Burundi and Zimbabwe, respectively. Both parents have been characterized as polyploid accessions with 36 chromosomes [33]. The CIAT 26146 x CIAT 16888 cross was performed by open pollination in the field, where an individual CIAT 26146 plant was surrounded by multiple clonal replicate plants of the apomictic male CIAT 16888. Preliminary analysis of SSR data revealed 12 of 124 progeny to be derived from accidental self-pollination of CIAT 26146. A further 10 progeny were excluded because they had severely distorted GBS results (*P* < 1 × 10^− 10^), with an extreme excess of heterozygote calls. The most likely cause of this distortion was determined to be mixing of leaf samples from neighboring plants during tissue collection. Therefore, only 102 total progeny were used for phenotyping, map construction, and subsequent analyses.

### Embryo sac analysis

Inflorescences for embryo sac analysis were harvested from the mapping population progeny in single plant plots at 2 m spacing at the CIAT research station in Popayán, Colombia (1760 masl; 2.4542° N, 76.6092° W)*.* Inflorescences were collected in the boot stage and fixed using formalin acetic acid (FAA) for 48 h. Samples were then stored in 70% ETOH, which was exchanged every 24 h for three days to remove residual formaldehyde.

The F_1_ progeny of the CIAT 26146 x CIAT 16888 mapping population were classified as apomictic or sexual by cytoembryological observation of methyl salicylate cleared pistils using differential interference contrast (DIC) microscopy [34, 35]. Abnormal (degenerated or ruptured) pistils are common in both apomictic and sexual *Brachiaria* plants [36]. The number of abnormal pistils was recorded for each of the progeny, and such pistils were excluded from further analyses. A minimum of ten pistils with normally developed embryo sacs were required to assess the reproductive mode of each of the progeny. Progeny with only Polygonum type embryo sac development were classified as sexual, while progeny with any pistils that had enlarged vacuolated nucellar cells or further Panicum type embryo sac development were classified as apomictic. A Chi Squared test was conducted to evaluate whether the population fit the expected segregation ratio for monogenic inheritance of the ASGR. Potential differences in the proportion of abnormal embryo sacs or the number of embryo sacs per pistil in apomictic and sexual progeny were assessed by analysis of variance (ANOVA) in SAS 9.2 (SAS Institute Inc., Cary, NC).

### Amplified fragment length polymorphism genotyping

Leaf tissue for DNA extractions for amplified fragment length polymorphism (AFLP) and SSR analysis was harvested from parents and progeny grown in greenhouse conditions at CIAT in Palmira, Colombia (1001 masl; 3.5833° N, 76.2500° W). Genomic DNA for AFLP genotyping was isolated from young leaves following the urea-phenol extraction protocol with slight modifications [37]. Evaluation of progeny with AFLP markers was performed following Vos et al. [38] with slight modifications. Five hundred ng of genomic DNA was digested with *Eco* RI/*Mse* I and ligated with *Eco* RI and *Mse* I adaptors. Pre-selective amplification of fragments was performed by one-nucleotide extension of C for the *Mse* I site and A for the *Eco* RI site, with a 2 min preliminary extension at 72 °C, followed by 25 cycles of 94 °C for 20 s, 56 °C for 30 s, and 72 °C for 2 min, and a 30 min final extension at 60 °C in a Gene Amp PCR System 9700 thermocycler (Life Technologies Japan, Tokyo, Japan). Selective amplification was performed with 64 primer combinations of FAM-labeled *Eco* RI primers and nonlabeled *Mse* I primers, with three-nucleotide extensions as described in the manufacturer’s instructions (Life Technologies Japan, Tokyo, Japan). Selective amplification was performed with a total of 35 cycles plus an initial denaturing step (20 s, 94 °C) and a final extension step (2 min, 72 °C). The annealing temperature for the first cycle was set at 66 °C, reduced by 1 °C for each of 10 subsequent cycles, and maintained at 56 °C for the final 25 cycles, as described by the AFLP Plant Mapping Protocol (Life Technologies). The duration of each annealing step was 30 s. Polymerase chain reactions (PCR) were performed using a Gene Amp PCR System 9700. Amplified DNA fragments were visualized using the ABI-3130 XL (Life Technologies) and GeneMapper v. 5.0 software. Bands that were present in only one of the parental genotypes and fit the 1:1 segregation ratio for presence and absence of bands in the progeny expected for single dose alleles (SDAs) were used in mapping.

### Simple sequence repeat marker development and genotyping

MSATCOMMANDER [39] was used to identify DNA sequences containing SSRs in Roche 454 FLX+ (Roche, Branford, CT) sequence data from the interspecific *Brachiaria* hybrid cv. Mulato II (CIAT 36087) and *B. humidicola* cv. Tully (CIAT 679). Primers were developed from SSR-containing sequences using Primer3Plus [40]. The names of SSR primers derived from the DNA sequences of Mulato II and Tully start with “B” and “BC,” respectively (Additional file 1: Table S1). These primers were tested for PCR amplification in Mulato II and Tully and the primers producing clear bands were screened for polymorphism in CIAT 26146 and CIAT 16888, the parents of the mapping population. Polymorphic PCR fragments were subsequently tested in the F_1_ mapping population using aliquots from the same DNA extractions used for AFLP genotyping. The SSR marker bands that fit the 1:1 ratio of heterozygotes and homozygotes expected for SDAs were used for mapping. Because only SDA markers were used in map construction, no attempts were made to score allele dosage in the progeny.

Forward primers were designed with a 5’-GGAAACAGCTATGACCAT M-13 reverse sequence tail for universal fluorescent labeling [41]. Polymerase chain reactions were carried out on a Biometra T1 thermocycler (Analytik Jena AG, Jena, Germany) in 10 μL final volume of reaction mixture. The reaction mix consisted of 10 ng genomic DNA, 1.0 μM forward and reverse primers, 200 μM dNTPs, 0.5 μL of AmpliTaq Gold ™ DNA polymerase (Applied Biosystems, Foster City, CA), and Buffer II. Initial denaturation was performed at 95 °C for 7 min. PCR amplifications were then conducted with 11 cycles of 95 °C for 1 m, 65 °C for 1 m, and 72 °C for 90 s, followed by 19 cycles of 95 °C for 1 m, 55 °C for 1 m, and 72 °C for 90 s, and a final elongation step at 72 °C for 10 min. Acrylamide gel electrophoresis was performed as described by Yamanaka et al. [42]. Products of PCR were visualized by GelRed ™ (Biotium, Fremont, CA) staining solution and the Pharos FX ™ scanner (Bio-Rad, Hercules, CA) and scored manually.

### Single nucleotide polymorphism marker development and genotyping

We evaluated a *B. humidicola* transcriptome reflecting gene expression under four different physiological stress conditions: high ammonium (1 mM); low nitrogen (110 μM) supplied in the form of both ammonium and nitrate; low phosphorus (1 μM); and high aluminum (200 μM) using adequate amounts of other nutrients in the form of low ionic strength nutrient solution, as described by Wenzl et al. [43]. The root and shoot tissue samples from each parent (CIAT 26146 and CIAT 16888) were collected and subsequently pooled and subjected to Illumina HiSeq2000 sequencing following paired-end library preparation with 500 bp average insert length. The four samples were barcoded and sequenced at Macrogen, obtaining a total of 233 million fragments for the four samples with a read length of 2 × 100, for a total production of 47 Gbp of sequence data. Although the read distribution was not completely even and 19 Gbp were assigned to the root tissue of CIAT 16888, at least 8.6 Gbp were obtained for each sample.

Illumina whole genome resequencing (WGS) of one of the progeny of the cross CIAT 26146 x CIAT 16888 was also performed. DNA was extracted as described for AFLP genotyping. Paired-end libraries with an average insert length of 500 bp were prepared and sequenced at the Yale Center for Genomic Analysis. This library was sequenced in a full Illumina HiSeq2000 lane. About 200 million fragments were obtained with a read length of 2 × 76 bp per fragment. The software SOAPdenovo v2.04 [44] was used to produce a draft assembly using 51 as k-mer size (-K option). The following parameters were set in the soapDeNovo configuration file: asm_flags = 3, rank = 1, pair_num_cutoff = 3 and map_len = 32. As expected, this produced a very fragmented assembly with 441,785 scaffolds and 2.45 million singleton contigs adding to 1.0 Mbp. Because the scaffold N50 was only 1003 bp, most genes were not expected to be assembled in single sequences. Hence, this draft was only used for reference-guided organization of the RNA-seq reads and to extract a DNA context for each single nucleotide polymorphism (SNP) selected to perform kompetitive allele specific PCR (KASP) genotyping. To discriminate single copy and repetitive regions in this assembly, raw WGS reads were aligned to the assembly using bowtie2 v2.2.3 [45] with default parameters except for the maximum number of alignments to retain for each read (−k), which was set to three and the maximum valid insert length (−X) which was set to 1000. Eighty-five percent of the reads aligned back to the assembly. Alignments were sorted by reference coordinates using Picard (https://broadinstitute.github.io/picard/). Then, the FindVariants command of the NGSEP pipeline v2.1.5 [46] was used with options -noRD, -noRP and -noSNVs to run only the clustering analysis of reads with multiple alignment to identify repetitive regions.

Because the RNA-seq assay presented above included reads from both CIAT 16888 and CIAT 26146, potential SNPs were identified by aligning the reads of the four samples (root and leave tissues from the two accessions) to the draft genome assembly with bowtie2, and calling variants using the NGSEP pipeline v2.1.5 [46]. The FindVariants command of NGSEP was called with parameters -noRep, −noRD and -noRP to call only SNVs and small indels. All other parameters were left with default values. After merging and genotyping predicted variants within the four RNA-seq samples in an single VCF file, suitable markers for genotyping were selected based on the following criteria: genotyping quality score (GQ ≥ 40) in all samples, only biallelic SNPs, consistent genotyping between tissues of the same individual, location in single copy regions, GC-content 40–65%, minimum distance to other variants of 40 bp, and existence of fewer than 30 unknown bp within a flanking region of 250 bp on either side of the SNP. KASP assays were designed based on the 279 transcriptome-derived SNPs considered suitable for genotyping that were homozygous in the draft *B. humidicola* genome assembly and heterozygous in CIAT 16888. This subset of markers was selected for KASP development to increase the chances of identifying SNPs in tight linkage with the ASGR. All KASP assays were used to genotype the full mapping population and parents. Marker reactions were conducted using LGC’s genotyping service (LGC Genomics, Beverly, MA) in a 4 μL reaction system including 2 μL low ROX KASP master mix, 0.106 μL of primer mix (0.318 μL of each primer at final concentration) and 2 μL of 10–25 ng/μl genomic DNA. The PCR conditions for the KASP assays were 94 °C for 15 min, followed by 10 cycles of touch down PCR from 68 °C to 60 °C with 0.8 °C decrease per cycle, and 30 cycles of 94 °C for 20 s and 57 °C for 1 min. PCR fluorescent endpoint readings were performed using the Light Cycler® 480 Real-Time PCR System (Roche, Germany).

### Genotyping with the ASGR-BBML specific primer pair p778/p779

The parents and progeny of the mapping population were also evaluated with p778/p779, a primer pair within the *Pennisetum squamulatum* (L.) R.Br. *ASGR-BABY BOOM-like* (*PsASGR-BBML*) candidate gene for the parthenogenesis component of apomixis [13]. Primer sequences and PCR conditions are reported in Worthington et al. [12].

### Genotyping by sequencing

Genotyping by sequencing was performed as described in Worthington et al. [12]. Briefly, libraries were prepared following Heffelfinger et al. [47] with the methylation-sensitive restriction enzyme *Hinc* II used for digestion. Sequencing was performed as 75 bp paired-end reads on the Illumina HiSeq 2500 in rapid run mode by the Yale Center for Genome Analysis (http://medicine.yale.edu/keck/ycga/index.aspx) following the manufacturer’s protocol.

The Tassel 3.0 universal network enabled analysis kit (UNEAK) pipeline was used for de novo SNP discovery and genotype calling [48]. A greater number of reads are required to make accurate genotypic calls in multisomic polyploid populations than diploid populations [49]. Therefore, we first made genotypic calls following recommendations for genotype calling in autotetraploids as described by Li et al. [49]. The threshold for calling a homozygote genotype was set at 11 or more reads of a single allele, while at least two reads of each allele and a minimum minor allele frequency greater than 0.10 was the requirement for calling a heterozygote. If neither condition was met, a missing data score was assigned. No attempt was made to call dosage and distinguish among the multiple genotypes possible for heterozygote individuals. We also performed genotype calling with more strict settings, requiring at least 17 reads of a single allele to call a homozygote genotype as recommended for autohexaploids [50]. Maps were constructed separately with each dataset. Because SNP order did not differ significantly between the maps constructed with the two datasets and the maps were more densely saturated with the original tetraploid settings, that data is presented in this manuscript.

The female parent of the mapping population (CIAT 26146) died prior to tissue collection for GBS. Fortunately, we had 12 progeny that were determined to be selfs of CIAT 26146 by SSR and AFLP analyses. These 12 selfed progeny were included in GBS sequencing with CIAT 16888 and the F_1_ progeny from the cross CIAT 26146 x CIAT 16888 and used to impute the female parent genotype for each SNP. CIAT 26146 was called as homozygous for markers that were homozygous in the same allelic state for all 12 selfed progeny. When the selfed progeny were either all heterozygous or segregating for a given SNP, CIAT 26146 was assumed to be heterozygous.

### Linkage map construction

Separate parental linkage maps of CIAT 26146 and CIAT 16888 were created in JoinMap 4.1 following the two-way pseudo-testcross strategy [51]. Markers that were heterozygous in only one parent and had a segregation ratio of less than 2:1 heterozygotes and homozygotes in the 102 progeny were classified as single dose alleles (SDA) and used in mapping. Single dose allele markers that were heterozygous in CIAT 26146 were used to construct the maternal linkage map, while SDAs heterozygous in CIAT 16888 were used to construct the paternal map. Markers with greater than 20% missing data were excluded from mapping.

Linkage groups were established using an initial threshold linkage logarithm of odds (LOD) score of 7.0. The Monte Carlo maximum likelihood (ML) mapping algorithm with default settings was used to determine marker order and distance within linkage groups. The initial CIAT 16888 linkage map had 38 linkage groups, but two pairs of linkage groups that clustered together at LOD 5.0 and 6.0 respectively were subsequently combined based on shared linkages with double-dose allele (DDA) markers and information on synteny with foxtail millet to form a total of 36 linkage groups. MapChart 2.1 [52] was used to produce charts of the genetic linkage maps.

### Synteny analysis and molecular karyotyping

Single nucleotide polymorphism markers aligned to unique positions in the foxtail millet genome were used to assign linkage groups to chromosomes and identify homologues. To perform the synteny analysis, consensus sequences of SDA tag pairs were extended using partially assembled 30x WGS sequence data from the diploid *B. ruziziensis* accession CIAT 26162 and a *B. humidicola* progeny from the cross CIAT 26146 x CIAT 16888 (Unpublished data). Tag reads were aligned to the contigs of the partially assembled genomes via NovoAlign (www.novocraft.com). Those contigs were used as the extended tag sequences and queried against the foxtail millet genome (https://phytozome.jgi.doe.gov/pz/portal.html#!info?alias=Org_Sitalica) [53] using the Basic Local Alignment Search Tool (BLAST) with a cutoff E-value of < 1 × 10^− 5^.

Meiotic associations between chromosomal regions with differing degrees of homology and homeology across the maternal and paternal genomes were assessed with high resolution molecular karyotyping [54] as described in Worthington et al. [12]. Each pair of mapped marker alleles was tested for segregation from the expected (1:1:1:1) ratio of individuals with both alleles present (1/1), one allele present (0/1 or 1/0), and neither allele present (0/0) for two alleles at a single homologous locus using Fisher’s exact test for count data (*P* < 0.05). Statistical analysis was conducted following Mason et al. [54] with minor modifications and heatmap figures were generated in R version 3.0.0 (The R Project for Statistical Computing).

Shared linkages with DDA markers were also used to identify homologous linkage groups in the maternal and paternal linkage maps. Single nucleotide polymorphism markers were classified as DDAs based on segregation at a 5:1 ratio of heterozygotes to homozygotes in the F_1_ progeny as expected for markers with tetrasomic inheritance or a 4:1 ratio as expected for markers with hexasomic inheritance according to χ^2^ tests (*P >* 0.05) [55]. Linkages between DDA and SDA markers from each parental map were first assessed using the preliminary grouping function of TetraploidMap under simplex-duplex linkages [56]. Because molecular karyotyping suggested that hexasomic inheritance predominated in CIAT 26146, SDA-DDA linkages identified in TetraploidMap for the maternal map were subsequently validated using χ^2^ tests (*P >* 0.05) for independence using expected hexasomic genotypic frequencies of independently segregating DDA and SDA markers (Table 1).

**Table 1:** Expected genotypic ratios for SDA-DDA linkages assuming tetrasomic or hexasomic inheritance^a^

	Tetrasomic	Hexasomic
AB^b^	1/2–1/6r	1/2–1/5r
A	1/6r	1/5r
B	1/3 + 1/6r	3/10 + 1/5r
0	1/6–1/6r	1/5–1/5r

^a^Expected genotypic ratio of progeny for single dose allele (SDA; A000 × 0000) and double dose allele (DDA; BB00 × 0000) markers linked in coupling

^b^‘AB’ indicates presence of segregating alleles in both SDA and DDA markers, ‘A’ indicates presence of the segregating allele for only the SDA marker, ‘B’ indicates presence of the segregating allele for only the DDA marker, ‘0’ indicates that neither segregating allele is present

## Results

### Analysis of reproductive mode

Eleven of the 102 F_1_ progeny never flowered over the course of the 18 months that the planting was established in Popayan and therefore could not be assessed for reproductive mode. A further 14 progeny could not be reliably diagnosed as apomictic or sexually reproducing because 74–100% of the pistils had aborted embryo sacs and it was not possible to reliably evaluate at least 10 pistils with normally developed embryo sacs. The remaining 77 F_1_ progeny which produced 10 or more normally developed embryo sacs segregated for reproductive mode at a 1:1 ratio (χ^2^ = 0.117, *P* = 0.73) (Table 2; Additional file 2: Table S2), as expected for the inheritance of a single dominant genetic factor. The progeny classified as sexually reproducing had only Polygonum type embryo sacs in all normally developed pistils, while the apomictic F_1_ hybrids showed at the minimum one pistil including at least one Panicum type embryo sac. The apomictic progeny had a range of normally developed pistils with Panicum type embryo sacs, Polygonum type embryo sacs, or both. The average proportion of Panicum type embryo sacs observed in the progeny classified as apomicts was 0.81 and ranged from 0.05–1.00 (Table 2; Additional file 2: Table S2). Only four of the 40 progeny classified as apomicts had 50% or more Polygonum type embryo sacs. While the sexual progeny exclusively had pistils with single Polygonum type embryo sacs, 40% of the evaluated pistils in the apomictic progeny had multiple embryo sacs (Table 2; Additional file 2: Table S2). The sexual progeny had significantly more pistils with aborted or abnormal embryo sacs than the apomictic progeny (*P <* 0.001) (Table 2; Additional file 2: Table S2).

**Table 2:** Reproductive mode of 77 F_1_ progeny in the CIAT 26146 x CIAT 16888 mapping population evaluated with embryo sac analysis

Reproductive mode	Sexual	Apomictic
No. of progeny	37	40
Proportion of pistils with Panicum-type embryo sacs		
Mean	0	0.81
Minimum	0	0.05
Maximum	0	1
Standard Deviation	0	0.21
Proportion of pistils with multiple embryo sacs		
Mean	0	0.40
Minimum	0	0
Maximum	0	1
Standard Deviation	0	0.27
Proportion of pistils with abnormal or aborted embryo sacs		
Mean	0.41	0.13
Minimum	0	0
Maximum	0.68	0.62
Standard Deviation	0.18	0.18

### Development of GBS and other markers

The segregating population was used to develop molecular markers and create dense parental linkage maps for CIAT 26146 and CIAT 16888. After quality filtering and processing with the UNEAK pipeline, a total of 51.7 million of the original 499.0 million sequencing reads (Additional file 3: Table S3) were assigned to 208,738 tag pair sites. After markers with over 20% missing data scores were removed, 6291 polymorphic GBS markers remained. Of these, 3475 markers (55%) were classified as SDAs, 2288 and 1187 of which were heterozygous in CIAT 26146 and CIAT 16888, respectively. A further 750 (12%) of markers in the dataset fit either a 5:1 or 4:1 segregation ratio (χ^2^, *P* < 0.05) and were classified as DDAs. Four hundred and fifty-four of the DDA markers were heterozygous in CIAT 26146, and 296 were heterozygous in CIAT 16888. UNEAK sequences of all mapped GBS-derived markers with variant alleles designated as ‘query’ and ‘hit’ according to Lu et al. [48] are given in Additional file 4: Table S4.

A total of 808 AFLP bands were classified as suitable for mapping because they were present in only one of the two parents and fit the 1:1 ratio of presence and absence expected for SDA markers. These bands were generated from 61 primer combinations, which produced between 1 and 47 SDA bands. One hundred and fifty-seven SSR bands produced from 114 primers fit the expected segregation ratio for SDA markers and were used in mapping. Of the 279 transcriptome-derived KASP assays, 160 (57%) were SDA markers suitable for mapping in this population. Primer sequences for mapped SSRs are given in Additional file 1: Table S1 and KASP primers are listed in Additional file 5: Table S5. A total of 2750 SDA markers were used in the development of the CIAT 26146 maternal haplotype map, including 2288 GBS-derived SNPs, 395 ALFP bands, 67 SSR bands, and 18 KASP markers. A further 1833 SDA markers, including 1187 GBS-derived SNPs, 413 ALFP bands, 90 SSRs, 142 KASP markers, and the ASGR specific marker p779/p780 were assigned to the CIAT 16888 paternal map.

### Genetic linkage maps

**Fig. 1:**
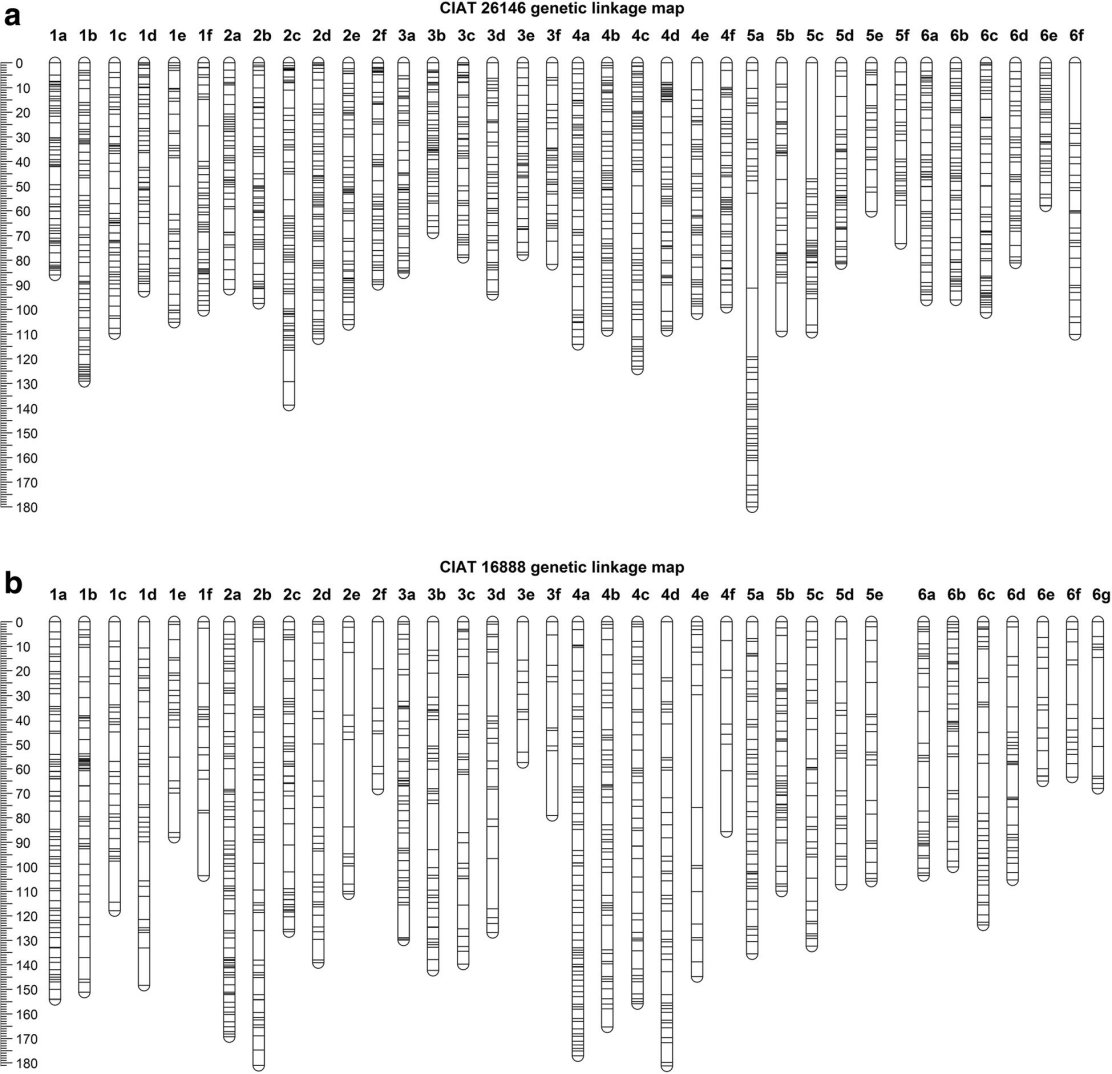
Parental linkage maps. The 36 linkage groups of the CIAT 26146 maternal linkage map (**a**) and the 36 linkage groups of the CIAT 16888 paternal linkage map (**b**). Homologous linkage groups were identified and assigned to chromosomes 1–6 based on synteny with foxtail millet (*S. italica*), molecular karyotyping, and shared linkages with double dose allele markers. Marker positions are expressed in centimorgans

The final CIAT 26146 maternal haplotype map had 2589 markers placed in 36 linkage groups with between 33 and 99 markers per linkage group (Table 3; Fig. 1a; Additional file 6: Table S6). The final map included 2180 GBS SNPs, 332 AFLPs, 61 SSRs, and 16 KASP markers. The total length of the CIAT 26146 haplotype map was 3558 cM, with an average marker density of one per 1.37 cM. The CIAT 16888 paternal haplotype map consisted of 1621 markers assigned to 36 linkage groups, with nine to 129 markers per linkage group (Table 3; Fig. 1b; Additional file 6: Table S6). The total map length was 4363 cM, with an average of one marker per 2.69 cM. The final paternal map included 1066 GBS SNPs, 352 AFLPs, 81 SSRs, 121 KASPs, and the indel p779/p780.

**Table 3:** CIAT 26146 and CIAT 16888 parental haplotype maps

Linkage Group	CIAT 26146		CIAT 16888	
	No. of markers	Length (cM)	No. of markers	Length (cM)
1a	97	86.0	94	154.1
1b	89	129.1	88	151.2
1c	82	109.8	52	117.9
1d	72	92.7	46	148.4
1e	70	105.2	28	88.0
1f	65	100.4	16	103.6
2a	93	91.8	129	169.3
2b	99	97.5	59	181.0
2c	96	138.7	63	126.5
2d	87	111.9	39	139.1
2e	86	106.1	17	111.0
2f	77	89.8	9	68.3
3a	72	85.1	63	129.9
3b	73	68.9	52	142.3
3c	62	78.9	41	139.7
3d	58	93.9	27	126.8
3e	58	77.8	12	57.4
3f	53	81.7	12	79.1
4a	93	114.2	98	177.1
4b	95	108.6	71	165.3
4c	84	124.2	57	155.8
4d	81	108.6	50	181.2
4e	77	101.7	20	144.8
4f	73	99.1	11	85.7
5a	82	180.0	65	135.5
5b	57	108.8	57	109.9
5c	57	109.3	42	132.4
5d	46	81.4	34	107.2
5e	41	60.3	29	105.8
5f	33	73.2	–	–
6a	82	96.2	53	103.5
6b	79	96.1	48	100.0
6c	74	101.3	51	123.7
6d	59	81.1	39	105.3
6e	51	58.0	17	65.0
6f	36	110.1	17	63.4
6 g	–	–	15	68.0
Total	2589	3557.7	1621	4362.9

### Synteny with foxtail millet

**Fig. 2:**
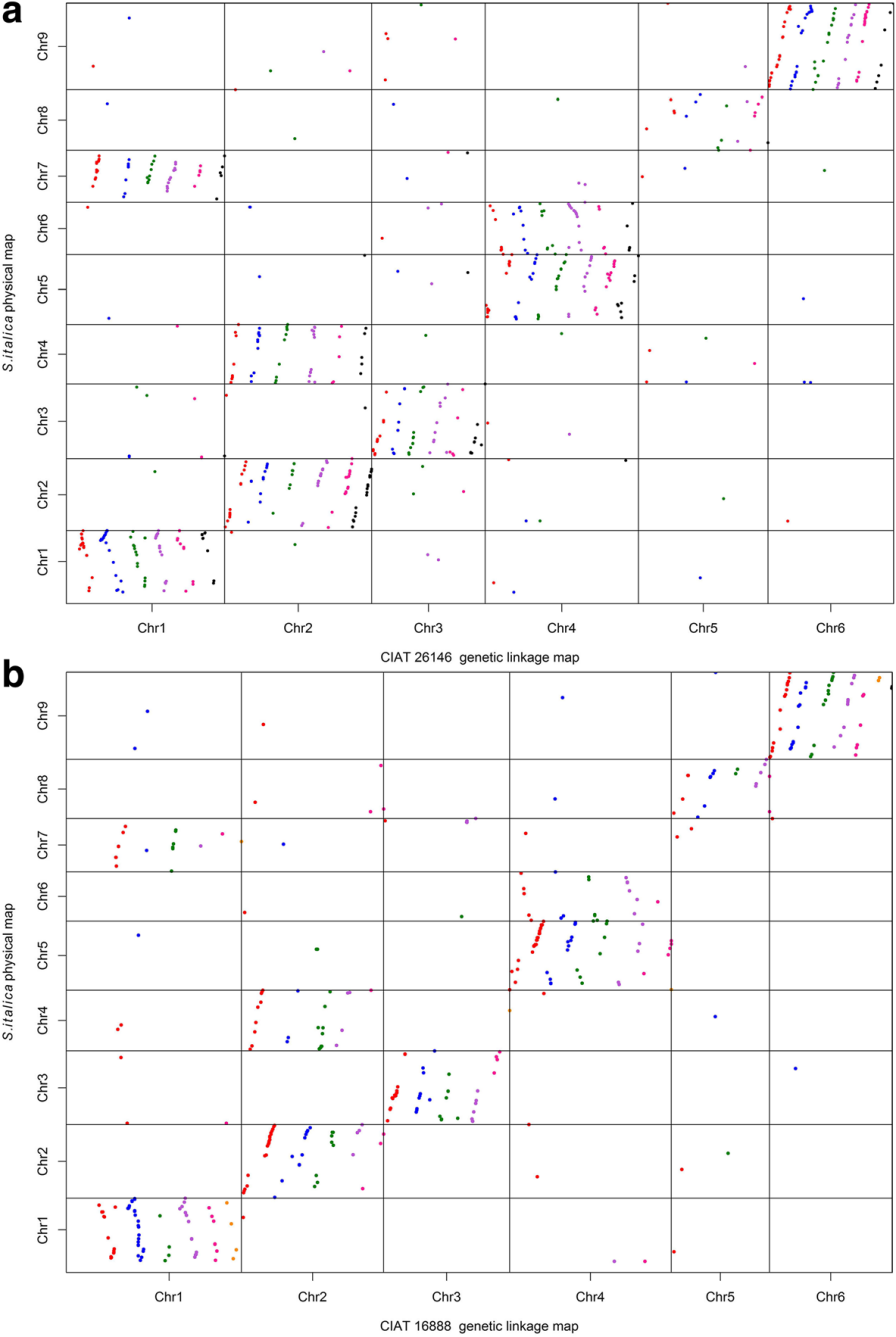
Synteny with Foxtail Millet. Alignment of markers mapped to the CIAT 26146 (**a**) and CIAT 16888 (**b**) genetic linkage maps with unique physical positions on the foxtail millet (*S. italica*) reference genome at a cutoff of *E-*value < 1 × 10^− 4^. Markers mapped to haplotypes a-g of each chromosome are represented with red, blue, green, purple, pink, black, and orange dots

Six hundred and eighty-eight (32%) of the GBS SNPs and seven (44%) of the KASP markers heterozygous in CIAT 26146 mapped to unique positions on the foxtail millet reference genome at a cutoff E-value of < 1 × 10^− 5^ (Fig. 2a; Additional file 6: Table S6). In the CIAT 16888 parental haplotype map, 356 (33%) GBS SNPs and 67 (55%) KASP markers mapped to unique positions on the foxtail millet reference genome (Fig. 2b; Additional file 6: Table S6). The distribution of markers with unique positions on the foxtail millet physical map was uneven across chromosomes, ranging from 187 markers mapped to unique positions on foxtail millet chromosome 9 to just 51 markers mapped to unique positions on chromosome 8 (Fig. 2; Additional file 6: Table S6).

Synteny analysis with foxtail millet indicated that the base chromosome number of *B. humidicola* is x = 6. Chromosomes 3, 5, and 6 of *B. humidicola* were highly collinear with foxtail millet chromosomes 3, 8, and 9, respectively. However, three pairs of foxtail millet chromosomes were fused in *B. humidicola*. *Brachiaria humidicola* chromosome 1 consisted of foxtail millet chromosomes 1 and 7, which remained intact and fused together at the proximal tips. Chromosome 2 of *B. humidicola* was composed of foxtail millet chromosome 4 sandwiched in between the two arms of chromosome 2, with the split on foxtail millet chromosome 2 occurring in the centromeric region between 15.4 and 19.3 Mbp. Likewise, *B. humidicola* chromosome 4 was composed of foxtail millet chromosome 5 split at the centromere between 19.7 and 22.5 Mbp, with intact chromosome 6 fused between the two arms (Fig. 2; Additional file 6: Table S6).

### Homologous linkage groups and preferential pairing

**Fig. 3:**
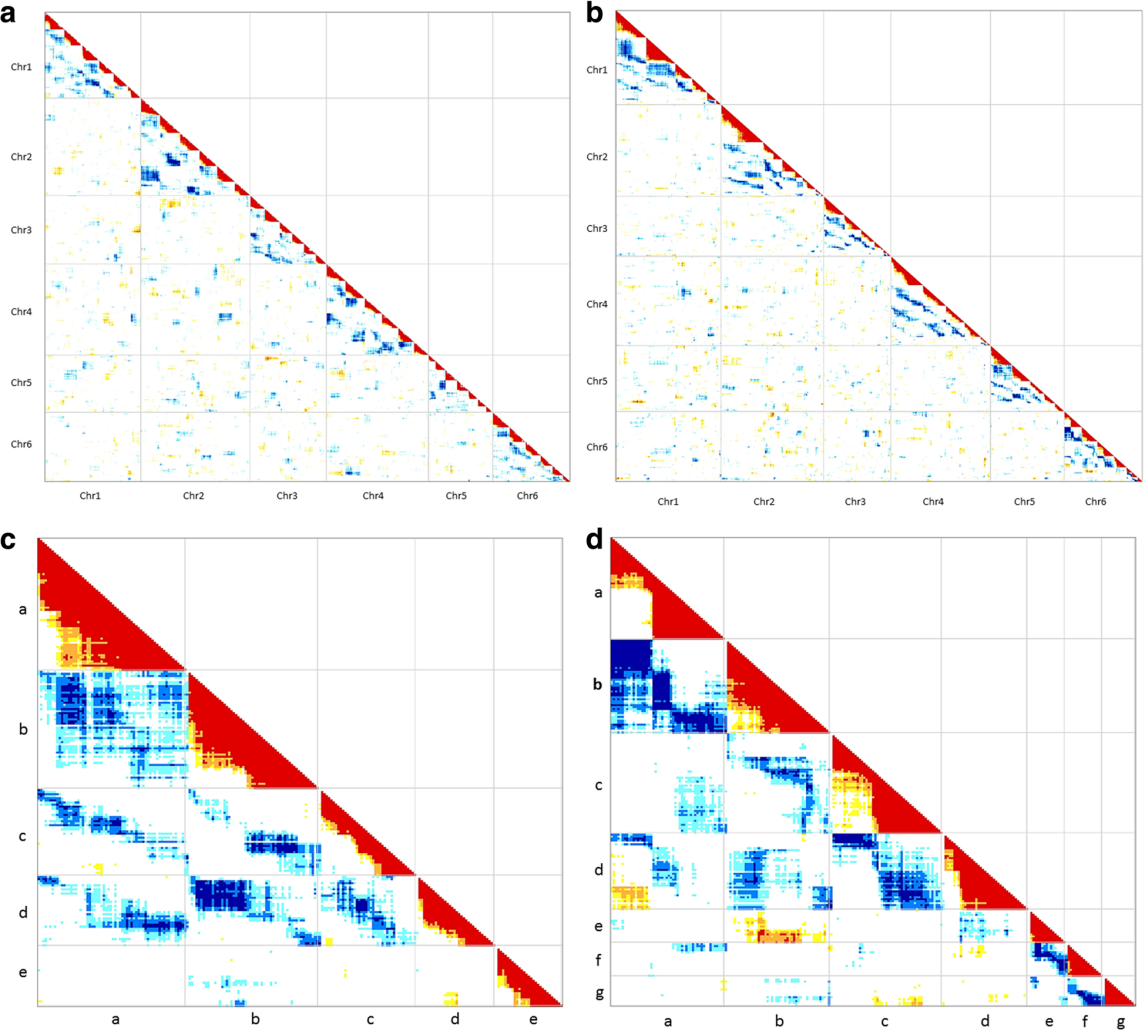
Molecular karyotyping of the parental linkage maps Linkage and segregation of markers in (**a**) the CIAT 26146 maternal haplotype map, (**b**) the CIAT 16888 paternal haplotype map, (**c**) CIAT 16888 chromosome 5 homologs a-e, and (**d**) CIAT 16888 chromosome 6 homologs a-g. SNP markers are arranged by their genetic position (not drawn to scale). Genetic regions with statistically significant linkage are indicated in red, orange, and yellow while regions with significant segregation are indicated with shades of blue. Light, medium, and dark blue indicate segregation significant at 0.001 < P < 0.05, 0.00001 < P  < 0.001, and P < 0.00005, respectively. Yellow, orange and red indicate linkage significant at 0.001 < P < 0.05, 0.00001 < P < 0.001, P < 0.00001, respectively

Six homologous linkage groups from the CIAT 26146 genetic map corresponding to each of the six base chromosomes of *B. humidicola* (Fig. 2a; Additional file 6: Table S6) were identified using synteny with foxtail millet and shared linkages with DDA markers. Of the 454 DDA markers that were heterozygous in CIAT 26146, 254 (56%) were linked in coupling with SDA markers from two homologous linkage groups of the maternal haplotype map (Table 4, Additional file 7: Table S7). The number of DDA markers placed on each chromosome ranged from 14 (chromosome 5) to 63 (chromosome 2). The DDA markers from each base chromosome were linked in coupling with each of the 15 possible pairs of homologs (a-f) at random (χ^2^, *P* > 0.05; Table 4), suggesting that there was no sub-genome differentiation in CIAT 26146. High-resolution molecular karyotyping also supported random assortment of the six homologous linkage groups of each chromosome in CIAT 26146 (Fig. 3a; Additional file 8: Table S8).

**Table 4:** DDA markers linked in coupling with parental haplotype linkage groups^a^

Haplotype pair	CIAT 26146						CIAT 16888					
	1	2	3	4	5	6	1	2	3	4	5	6
a,b	5	6	1	6	2	1	6	0	1	2	1	2
a,c	2	4	1	1	1	5	2	7	0	6	0	3
a,d	3	2	0	5	2	0	3	9	1	6	1	6
b,c	1	1	1	6	0	3	8	15	8	18	5	5
b,d	7	3	1	3	1	3	5	6	6	12	2	3
c,d	1	2	5	1	1	4	7	8	6	18	2	6
a,e	1	1	4	2	2	2	0	0	0	0	0	0
a,f	7	4	0	4	0	0	0	0	0	0	–	0
a,g	–^c^	–	–	–	–	–	–	–	–	–	–	0
b,e	3	6	2	4	1	5	0	0	0	0	1	0
b,f	3	4	3	0	1	4	0	1	0	0	–	0
b,g	–	–	–	–	–	–	–	–	–	–	–	0
c,e	4	6	1	4	0	5	0	0	0	0	0	0
c,f	1	10	4	6	1	3	0	0	0	0	–	0
c,g	–	–	–	–	–	–	–	–	–	–	–	0
d,e	2	3	3	6	1	1	0	0	0	0	0	0
d,f	1	6	3	1	1	6	0	0	0	0	–	0
d,g	–	–	–	–	–	–	–	–	–	–	–	0
e,f	4	5	5	3	0	4	8	0	0	9	–	1
e,g	–	–	–	–	–	–	–	–	–	–	–	0
f,g	–	–	–	–	–	–	–	–	–	–	–	3
total	45	63	34	52	14	46	39	46	22	71	12	29
*P* ^b^	0.13	0.16	0.2	0.22	0.92	0.28	< 0.001	< 0.001	< 0.001	< 0.001	0.035	< 0.001

^a^Number of double dose alleles (DDAs) linked in coupling with each possible pair of SDA haplotypes (a-g) of the six base chromosomes of *B. humidicola* on the CIAT 26146 and CIAT 16888 genetic maps

^b^*P* values for χ^2^ tests of random distribution of shared DDA linkages assuming there is no differentiation of sub-genomes

^c^Indicates that this haplotype pair does not exist for the given chromosome

The 36 linkage groups of the CIAT 16888 paternal haplotype map were first assigned to chromosomes based on synteny with foxtail millet and molecular karyotyping results. Synteny analysis showed six linkage groups corresponded to *B. humidicola* chromosome 1 (a-f), five linkage groups corresponded to chromosomes 2–5 (a-e), and seven linkage groups corresponded to chromosome 6 (a-g) (Fig. 2b; Additional file 6: Table S6). The remaining three linkage groups were assigned to chromosomes 2, 3, and 4 based on segregation patterns revealed in molecular karyotyping analysis (Fig. 3b; Additional file 9: Table S9). Molecular karyotyping indicated that there were two sets of preferentially pairing linkage groups for each chromosome of CIAT 16888 (Fig. 3b; Additional file 9: Table S9). Four homologous linkage groups (a-d) of each chromosome paired at random. On chromosomes 1–4, the remaining two linkage groups (e-f) paired preferentially with each other, while the fifth homolog (e) of chromosome 5 showed no significant segregation with any other linkage group (Fig. 3c). The three remaining linkage groups (e-g) of chromosome 6 showed significant segregation with each other, though not with homologs (a-d) (Fig. 3d). There was insufficient linkage to combine any of the seven linkage groups of chromosome 6, even at a linkage LOD of 2.0, suggesting that the unbalanced number of linkage groups assigned to each chromosome may be due to compensated aneuploidy rather than insufficient marker density.

**Fig. 4:**
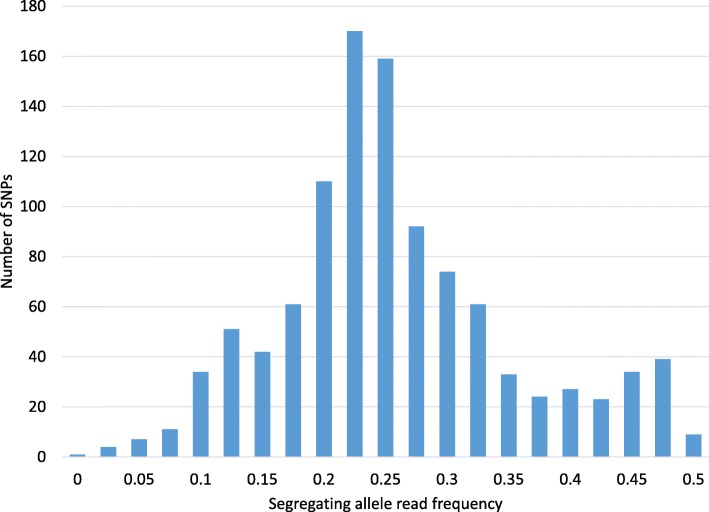
Read Frequency in CIAT 16888. Relative depth of coverage (read frequency) of the segregating allele in heterozygous loci in the CIAT 16888 paternal linkage map

Shared linkages with DDA markers and segregating allele read frequency showed further evidence of sub-genome differentiation in CIAT 16888. Two hundred and nineteen (80%) of the 296 DDA markers heterozygous in CIAT 16888 were linked in coupling with two linkage groups corresponding to the same base chromosome from the paternal haplotype map. Between 12 (chromosome 5) and 71 (chromosome 4) DDA markers were in linkage with SDA markers from each chromosome. In contrast to the random distribution of shared DDA linkages among homologs in CIAT 26146, significantly more DDA markers in CIAT 16888 had shared linkages with just four (a-d) homologous linkage groups of each chromosome than would be expected by chance (χ^2^, *P* < 0.05, Table 4; Additional file 7: Table S7). A strong peak in segregating allele read frequency (ratio of reads for the segregating allele to total reads) in GBS SDA markers was observed around 0.25 in the CIAT 16888 haplotype map as expected for an autotetraploid, with lesser peaks at 0.125 and 0.5 (Fig. 4). This finding suggests that while some SNPs were present on all homologs, the majority of SNPs existed in only one of two differentiated sub-genomes of CIAT 16888.

### Genetic mapping of the ASGR

**Fig. 5:**
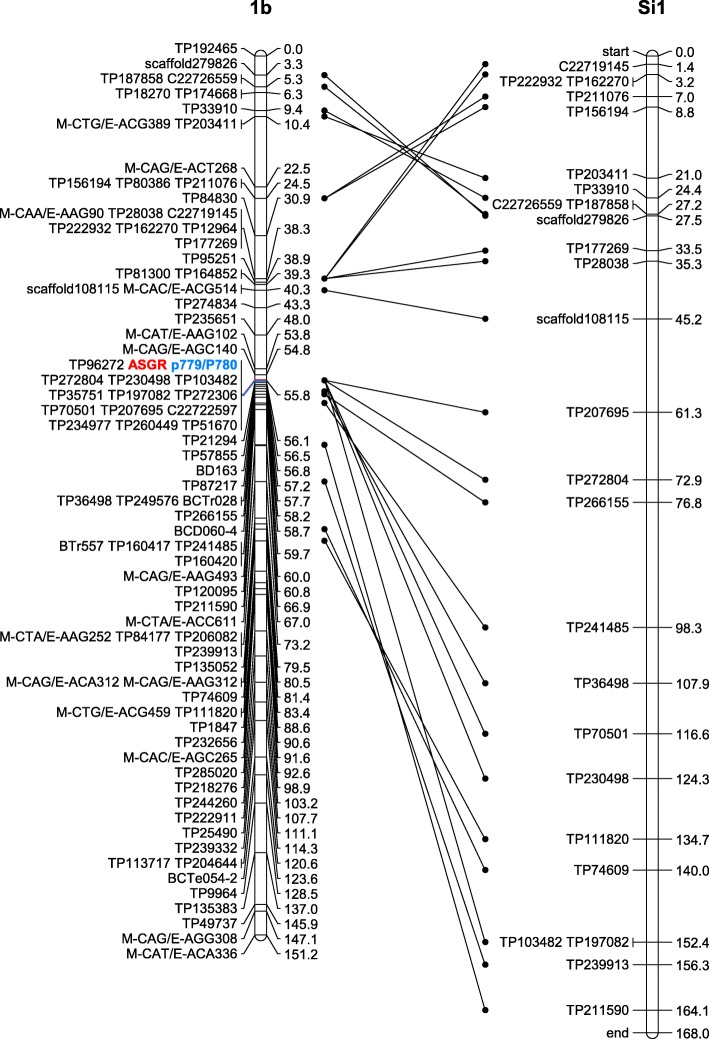
Comparison of the ASGR carrier chromosome with the physicial map of *S. italica* chromosome 1. Synteny between CIAT 16888 linkage group 1b, the carrier chromosome of the apospory specific genomic region (ASGR), and foxtail millet (*S. italica*) chromosome 1. The ASGR and the ASGR-specific amplicon from p778/p779 are in red and blue bold font respectively. Genetic positions are given in centimorgans. The physical map of foxtail millet chromosome 1 has been inverted to facilitate visualization of synteny and each unit of the physical map represents 2.5 × 10^5^ bp

The ASGR was mapped to position 55.8 cM of CIAT 16888 linkage group 1b (Fig. 5; Additional file 6: Table S6). A total of 14 markers, including the *ASGR-BBML*-specific marker p779/p780, one transcriptome-derived KASP marker, and 12 GBS markers, were in perfect linkage with the ASGR. Although *B. humidicola* chromosome 1 is a composite of foxtail millet chromosomes 1 and 7, the ASGR mapped to a region that is clearly syntenous with foxtail millet chromosome 1. Six of the GBS markers in perfect linkage with the ASGR mapped to unique positions on foxtail millet chromosome 1 ranging from 3.9 to 26.7 Mbp. These markers included TP197082 and TP103482 (3.9 Mbp), TP230498 (10.9 Mbp), TP70501 (12.9 Mbp), TP272804 (23.8 Mbp), and TP207695 (26.7 Mbp). This physically large region includes the centromere of foxtail millet chromosome 1 [53]. The ASGR was flanked at 1.0 cM proximal by the AFLP marker M-CAG/E-AGC140 and at 0.3 cM distal by the GBS marker TP21294. The closest flanking markers that mapped to unique positions on foxtail millet chromosome 1 were the transcriptome-derived KASP marker scaffold108115 (30.8 Mbp) at 40.30 cM and the GBS marker TP36498 (15.0 Mbp) at 57.7 cM.

## Discussion

With over 4210 total mapped markers, the parental maps reported here have over ten times the marker density of the best existing map of *B. humidicola* [26]. Thirty-six linkage groups were identified for each parental map, as expected based on chromosome counts and flow cytometry [28, 33]. Dense maps of an interspecific *B. decumbens* x *B. ruziziensis* mapping population showed that both species had a base chromosome number of x = 9 and a high degree of collinearity with foxtail millet, the closest relative of *Brachiaria* with a publically available reference genome [12]. The only major structural rearrangements discovered between those species and foxtail millet were a reciprocal translocation between the proximal and distal tips of chromosomes 3 and 7 and inversions on chromosomes 1, 3, 5, and 6. In contrast, synteny analysis of the *B. humidicola* parental maps with the foxtail millet physical map showed clearly that there were six base chromosomes in this species. This finding is supported by previous cytogenetic evidence for a base chromosome number of x = 6 in *B. humidicola* and *B. dictyoneura* including the presence of hexavalents and other higher order multivalents in genebank accessions with 2n = 36 and higher chromosome numbers [23, 26, 57, 58].

Chromosomes 3, 5, and 6 of *B. humidicola* had a high degree of synteny with chromosomes 3, 8, and 9 of foxtail millet, *B. decumbens*, and *B. ruziziensis* (Fig. 2; Additional file 6: Table S6). The lower base chromosome number of *B. humidicola* relative to *B. decumbens* and *B. ruziziensis* was accounted for by the fusion of three pairs of chromosomes (*S. italica* chromosomes 1 and 7 = *B. humidicola* chromosome 1; *S. italica* chromosomes 2 and 4 = *B. humidicola* chromosome 2; and *S. italica* chromosomes 5 and 6 = *B. humidicola* chromosome 4). This fusion of chromosomes is consistent with the large chromosome size of *B. humidicola* relative to other *Brachiaria* species [59]. The strongly conserved synteny between foxtail millet and *B. decumbens* and *B. ruziziensis* suggests that the *Brachiaria* agamic complex species (*B. brizantha*, *B. decumbens*, and *B. ruziziensis*) or other x = 9 *Brachiaria* species are likely ancestral to *B. humidicola.*

### Meiotic pairing

Vigna et al. [26] argued for a recent allopolyploid (AABBBB) origin of CIAT 26146 (H031) and the apomictic cultivar ‘Tupi’ based on the presence of two nucleoli of differing sizes in some meiocytes, the high frequency of disomic and tetrasomic chromosome associations at diakinesis, and differences in sizes of preferentially pairing chromosomes. They suggested that hexaploid *B. humidicola* likely originated from a cross between a sexual diploid female (2n = 2x = 12, genome A) and a tetraploid apomictic male (2n = 4x = 24, genome B) that produced a triploid progeny (2n = 3x = 18, ABB) which experienced spontaneous chromosome doubling (2*n* = 6x = 36, AABBBB). Alternatively, they suggest that the triploid progenitor could have also originated from the cross of two diploid parents (2n = 2x = 12), one contributing a reduced gamete (n = 6, genome A) and the other contributing an unreduced gamete (*n* = 12, genome B).

Our results from molecular karyotyping, segregating allele read frequency, and DDA-SDA linkage analysis support allopolyploidy (AABBBB) and preferential pairing of sub-genomes in the apomictic male parent CIAT 16888 (Fig. 3b; Fig. 4; Table 4). However, our DDA-SDA linkage results showed no evidence of subgenome differentiation in the sexual female parent (CIAT 26146) (Table 4). Molecular karyotyping analysis also indicated that pairing among the six homologs of each base chromosome in CIAT 26146 occurred at random (Fig. 3a). These findings support hexasomic segregation and possibly autohexaploidy (BBBBBB) in CIAT 26146.

While it is unexpected that two accessions of the same species would have such different meiotic behavior, two detailed phylogenies of *B. humidicola* have reported that CIAT 26146 is very distantly related to all the apomictic accessions held in the CIAT and EMBRAPA germplasm collections based on STRUCTURE, UPGMA, and NJ analyses [17, 33]. The prevalence of bivalent and tetravalent chromosome associations in diakinesis in CIAT 26146 is not necessarily evidence against autopolyploidy or hexasomic segregation. Bivalents associations predominate in a number of autotetraploid species, including alfalfa (*Medicago sativa*) [60]. If the sexual and apomictic accessions of *B. humidicola* do indeed have different genomic composition (autopolyploid versus allopolyploid), then CIAT 26146 may need to be classified as a separate species or subspecies.

The origin and ancestry of CIAT 26146 remains unknown. Of the 66 *B. humidicola* accessions maintained in the CIAT *Brachiaria* collection, CIAT 26146 is the only sexually reproducing genotype. Furthermore, CIAT 26146 is the only documented naturally occurring polyploid sexual genotype in the entire collection of 601 *Brachiaria* genotypes maintained by CIAT. Eight other *Brachiaria* species, including *B. bovonei* (Chiov.) Robyns, *B. brevispicata* (Rendle) Stapf, *B. dictyoneura* (Fig. et De Not.) Stapf, *B. jubata* (Fig. et De Not.) Stapf, *B. platynota* (K. Schum.) Robyns, *B. reticulata* Stapf, *B. stigmatisata* (Mez) Stapf, and *B. subulifolia* (Mez) Clayton, were assigned to taxonomic group six with *B. humidicola* based on panicle and inflorescence morphology [14]. Of these species, only *B. humidicola* and *B. dictyoneura* have been used as commercial forages. The noncommercial species of *Brachiaria* are poorly represented in the CIAT and EMBRAPA germplasm collections relative to commercial species and have been studied less intensively. Comparative genomics and cytological studies including genome in situ hybridization (GISH) with other taxonomic group six species is needed to elucidate the origin, divergence, and evolution of CIAT 26146 and the apomictic *B. humidicola* accessions.

Although 36 linkage groups were found in the apomictic parent CIAT 16888 as expected for a euploid hexaploid genotype (2n = 6x = 36), molecular karyotyping analysis indicated that there were only five linkage groups for chromosome 5 (a-e) and seven linkage groups for chromosome 6 (a-g). This evidence for compensated aneuploidy in the apomict CIAT 16888 is not too surprising given that meiotic errors and unbalanced gametes occur with a high degree of frequency in *B. humidicola* [26, 61]. Furthermore, the high frequency of septaploids (2n = 7x = 42) and nonaploids (2n = 9x = 54) in the CIAT and EMBRAPA *B. humidicola* collections indicate that this species may be tolerant of aneuploidy [28, 33]. Compensated aneuploidy has been documented in the recently formed natural allopolyploid species *Tragopogon miscellus* [62] and in experimental neoallopolyploids in the *Triticum* and *Brassica* genera [63, 64]. Evidence for segmental allopolyploidy, including frequent non-homologous chromosome pairing, has been documented in *B. decumbens* [12]. Apomixis has been suggested as a form of meiotic restitution that arrests the process of diploidization and allows polyploid species to remain in a neopolyploid state indefinitely [12]. This evidence of compensated aneuploidy in CIAT 16888 supports the theory that apomixis promotes fertility in meiotically unstable neopolyploid grasses.

The evidence of possible compensated aneuploidy in CIAT 16888 suggests that it may not be a good choice as a male parent in breeding programs. Caryopsis formation rarely exceeds 30% in *Brachiaria* [65]. Low seed set is a persistent limitation in *Brachiaria*, and seed yield potential impacts whether a new variety can be profitably produced and distributed to farmers. Seed production is especially difficult in *B. humidicola*; seed of *B. humidicola* cultivars ‘common’ and Llanero was over twice the cost of *B. brizantha* [66]. Low seed yield in *B. humidicola* may be associated with poor pollen viability. *Brachiaria* grasses are pseudogamous [3], which means that the secondary nuclei of apomictic embryo-sacs must be fertilized with viable pollen gametes for normal endosperm development and seed production. Abnormal tetrad frequency was correlated with non-viability of pollen grains [67]. An aneuploid apomictic pollen donor is likely to contribute unbalanced gametes to a high frequency of its progeny. While some of these aneuploid progeny may produce acceptable forage biomass, their success as cultivars could be limited by poor seed yield.

### Conservation of the ASGR in the Paniceae and translocation to an alternate carrier chromosome

As expected, the inheritance of apomixis in the CIAT 26146 x CIAT 16888 mapping population fit the segregation ratio for a single dominant Mendelian factor. The ASGR mapped to position 55.8 cM of CIAT 16888 linkage group 1b (Fig. 5; Additional file 6: Table S6), a region syntenic with foxtail millet chromosome 1. The number of markers in perfect linkage with the ASGR and the large physical distance between them suggests that the ASGR is located in a region of suppressed recombination. Evidence of reduced recombination around the ASGR has also been reported in *P. squamulatum* [4], *P. notatum* and *P. simplex* [5–9], and *B. decumbens* [12].

The location of the ASGR on linkage group 1b of *B. humidicola* is surprising given that the ASGR was mapped to position 42.5 cM of *B. decumbens* linkage group 5c, a region syntenous with foxtail millet chromosome 5 [12]. On the other hand, studies in *B. brizantha* have found that the ASGR was linked to RFLP probes designed from rice chromosome 2 and maize chromosome 5 [68, 69]. Both rice chromosome 2 and maize chromosome 5 are mostly syntenic with foxtail millet chromosome 1 [53]. This suggests that *B. humidicola* may have an ASGR carrier chromosome more closely related to *B. brizantha* than *B. decumbens.* The *P. squamulatum* ASGR-carrier chromosome was found to be collinear with foxtail millet chromosome 2 and sorghum chromosome 2 by fluorescence in situ hybridization (FISH) and in silico transcript mapping [70].

Three different ASGR-carrier chromosomes (collinear with foxtail millet chromosomes 1, 2, and 5) have been identified in the Paniceae to date. The implication of different ASGR-carrier chromosomes has been cited as evidence for independent evolution of apomixis in multiple grass species [71]. However, the perfect linkage of the *ASGR-BBML* specific primers p779/p780 with reproductive mode in independent mapping populations of *B. decumbens*, *B. humidicola*, and *P. squamulatum* indicates that apomixis more likely evolved as a single event and was spread to other species through hybridization or phylogenetic diversification [12, 13]. Comparative genomics with ASGR-linked BACs in *Cenchrus* and *Pennisetum* also support the hypothesis of a common origin for aposporous apomixis in the Paniceae tribe [13, 72].

## Conclusions

The development of dense molecular maps in hexaploid *B. humidicola* has provided further support for cytogenetic evidence indicating a base chromosome number of six in this species. Analysis of SDA-DDA linkages, synteny with foxtail millet, and molecular karyotyping all supported previous evidence of allopolyploid origin in apomictic *B. humidicola.* However, these same analyses indicated that there was no significant differentiation of subgenomes or preferential chromosome pairing in the sexually reproducing female parent, CIAT 26146. The evidence of compensated aneuploidy in the apomictic male parent, CIAT 16888, supports the theory that apomixis acts as a form of meiotic restitution that allows unstable polyploid species to remain in a neopolyploid state indefinitely. Our data show that the *ASGR-BBML* specific amplicon from p779/p780 was in full linkage with the ASGR in the F_1_ *B. humidicola* mapping population despite its location on a different carrier chromosome than previously identified in *B. decumbens*. These results provide further evidence of conservation of *ASGR-BBML* gene sequences across the Paniceae and support their postulation as candidate genes for the parthenogenesis component of apomixis.

## Additional files

Additional file 1: **Table S1.** Simple sequence repeat (SSR) primers used in the study. (XLSX 18 kb)

Additional file 2: **Table S2.** Detailed results of embryo sac analysis. (XLSX 14 kb)

Additional file 3: **Table S3.** Depth of genotyping-by-sequencing read coverage in the CIAT 26146 x CIAT 16888 mapping population. (XLSX 13 kb)

Additional file 4: **Table S4.** UNEAK sequences of GBS derived markers. (XLSX 259 kb)

Additional file 5: **Table S5.** Primer sequences for Kompetitive allele specific PCR (KASP) markers in the CIAT 26146 and CIAT 16888 parental linkage maps. (XLSX 17 kb)

Additional file 6: **Table S6.** Single dose allele marker positions in the CIAT 26146 and CIAT 16888 parental linkage maps, physical positions on the *S. italica* reference genome, deviations from the expected 1:1 ratio of heterozygotes to homozygotes in the F_1_ progeny, and genotype scores in the progeny. (XLSX 1944 kb)

Additional file 7: **Table S7.** Double dose allele markers and linkages with SDA haplotypes in the CIAT 26146 and CIAT 16888 parental maps. (XLSX 216 kb)

Additional file 8: **Table S8.** Segregation of alleles within the CIAT 26146 genetic map. Marker pairs with statistically significant segregation and co-segregation interactions based on Fisher’s exact test for count data are respectively colored orange and blue. (XLSX 50402 kb)

Additional file 9: **Table S9.** Segregation of alleles within the CIAT 16888 genetic map. Marker pairs with statistically significant segregation and co-segregation interactions based on Fisher’s exact test for count data are respectively colored orange and blue. (XLSX 15261 kb)

## Abbreviations

AFLP: Amplified fragment length polymorphism
ANOVA: Analysis of variance
ASGR: Apospory-specific genomic region
*ASGR-BBML*: *ASGR-BABY BOOM-like*
*BBM*: *BABY BOOM*
DDA: Double-dose allele
DIC: Differential interference contrast
EBC: EMBRAPA Beef Cattle
GBS: Genotyping-by-sequencing
KASP: Kompetitive allele specific PCR
NJ: Neighbor joining
PCR: Polymerase chain reactions
RAPD: Random amplified polymorphic DNA
SCAR: Sequenced characterized amplified region
SDA: Single-dose allele
SNP: Single nucleotide polymorphism
SSR: Simple sequence repeat
UNEAK: Universal network enabled analysis kit
UPGMA: Unweighted pair group method with arithmetic mean
WGS: Whole genome resequencing

## References

[1] Ozias-Akins P, van Dijk PJ (2007). Mendelian genetics of apomixis in plants. Annu Rev Genet.

[2] Hand ML, Koltunow AMG (2014). The genetic control of apomixis: asexual seed formation. Genetics.

[3] Barcaccia G, Albertini E (2013). Apomixis in plant reproduction: a novel perspective on an old dilemma. Plant Reprod.

[4] Ozias-Akins P, Roche D, Hanna WW. Tight clustering and hemizygosity of apomixis-linked molecular markers in *Pennisetum squamulatum* implies genetic control of apospory by a divergent locus that may have no allelic form in sexual genotypes. Proc Natl Acad Sci U S A. 1998;95:5127–32. 10.1073/pnas.95.9.5127 PMC20225 9560240

[5] Stein J, Quarin CL, Martínez EJ, Pessino SC, Ortiz JPA. Tetraploid races of *Paspalum notatum* show polysomic inheritance and preferential chromosome pairing around the apospory-controlling locus. Theor Appl Genet. 2004;109:186–91. 10.1007/s00122-004-1614-z 14985979

[6] Stein J, Pessino SC, Martínez EJ, Rodriguez MP, Siena LA, Quarin CL, et al. A genetic map of tetraploid *Paspalum notatum* Flügge (bahiagrass) based on single-dose molecular markers. Mol Breed. 2007;20:153–66.

[7] Pupilli F, Martinez EJ, Busti A, Calderini O, Quarin CL, Arcioni S. Comparative mapping reveals partial conservation of synteny at the apomixis locus in *Paspalum* spp. Mol Gen Genomics. 2004;270:539–48. 10.1007/s00438-003-0949-5 14648202

[8] Podio M, Felitti SA, Siena LA, Delgado L, Mancini M, Seijo JG, et al. Characterization and expression analysis of SOMATIC EMBRYOGENESIS RECEPTOR KINASE (SERK) genes in sexual and apomictic *Paspalum notatum*. Plant Mol Biol. 2014;84:479–95. 10.1007/s11103-013-0146-9 24146222

[9] Calderini O, Chang SB, De Jong H, Busti A, Paolocci F, Arcioni S, et al. Molecular cytogenetics and DNA sequence analysis of an apomixis-linked BAC in *Paspalum simplex* reveal a non pericentromere location and partial microcolinearity with rice. Theor Appl Genet. 2006;112:1179–91. 10.1007/s00122-006-0220-7 16463157

[10] Conner JA, Mookkan M, Huo H, Chae K, Ozias-Akins P (2015). A parthenogenesis gene of apomict origin elicits embryo formation from unfertilized eggs in a sexual plant. Proc Natl Acad Sci U S A.

[11] Boutilier K (2002). Ectopic expression of BABY BOOM triggers a conversion from vegetative to embryonic growth. Plant Cell Online.

[12] Worthington M, Heffelfinger C, Bernal D, Quintero C, Zapata YP, Perez JG, et al. A parthenogenesis gene candidate and evidence for segmental allopolyploidy in apomictic *Brachiaria decumbens*. Genetics. 2016;203:1117–32. 10.1534/genetics.116.190314 PMC4937464 27206716

[13] Akiyama Y, Goel S, Conner JA, Hanna WW, Yamada-Akiyama H, Ozias-Akins P. Evolution of the apomixis transmitting chromosome in *Pennisetum*. BMC Evol Biol. 2011;11:289 Available from: http://www.biomedcentral.com/1471-2148/11/289. BioMed Central Ltd. 10.1186/1471-2148-11-289 PMC3198970 21975191

[14] Renvoize SA, Clayton WD, Kabuye CHS. Morphology, taxonomy and natural distribution of *Brachiaria* (Trin.) Griseb. In: Miles JW, Maass BL, Valle CB, editors. *Brachiaria*: Biology, Agronomy, and Improvement. Palmira: CIAT; 1996. p. 1–15.

[15] Keller-Grein G, Maass BL, Hanson J. Natural variation in *Brachiaria* and existing germplasm collections. In: Miles JW, Maass BL, Valle CB, editors. *Brachiaria*: Biology, Agronomy, and Improvement. Palmira: CIAT; 1996. p. 16–35.

[16] Pessoa-filho M, Martins AM, Ferreira ME. Molecular dating of phylogenetic divergence between *Urochloa* species based on complete chloroplast genomes. BMC Genomics. 2017;18:516. 10.1186/s12864-017-3904-2 PMC5499013 28683832

[17] Triviño NJ, Perez JG, Recio ME, Ebina M, Yamanaka N, Tsuruta S, et al. Genetic diversity and population structure of *Brachiaria* species and breeding populations. Crop Sci. 2017;57:2633-44.

[18] Valle CB, Savidan YH. Genetics, cytogenetics, and reproductive biology of *Brachiaria*. In: Miles JW, Maass BL, Valle CB, editors. *Brachiaria*: Biology, Agronomy, and Improvement. Palmira: CIAT; 1996. p. 147–63.

[19] Mendes-Bonato AB, Risso-Pascotto C, Pagliarini MS, Do Valle CB. Chromosome number and meiotic behaviour in *Brachiaria jubata* (Gramineae). J Genet. 2006;85:83–7. 10.1007/BF02728976 16809846

[20] Mendes-Bonato AB, Pagliarini MS, Forli F, Valle CB, Penteado MIO. Chromosome numbers and microsporogenesis in *Brachiaria brizantha* (Gramineae). Euphytica. 2002;125:419–25.

[21] Risso-Pascotto C, Pagliarini MS, Do Valle CB. Microsporogenesis in *Brachiaria bovonei* (Chiov.) Robyns and *B. subulifolia* (Mez) Clayton (Poaceae). Sci Agric. 2009;66:691–6.

[22] Risso-Pascotto C, Mendes DV, Silva N, Pagliarini MS, Valle CB. Evidence of allopolyploidy in *Brachiaria brizantha* (Poaceae: Paniceae) through chromosome arrangement at metaphase plate during microsporogenesis. Genet Mol Res. 2006;5:797–803. 17183487

[23] Boldrini KR, Pagliarini MS, Valle CB. Meiotic behavior of a nonaploid accession endorses x = 6 for *Brachiaria humidicola* (Poaceae). Genet Mol Res. 2009;8:1444–50. 10.4238/vol8-4gmr679 20013658

[24] Boldrini KR, Pagliarini MS, Valle CB. Evidence of natural hybridization in *Brachiaria humidicola *(Rendle) Schweick. (Poaceae: Panicoideae: Paniceae). J Genet. 2010;89:91–4. 10.1007/s12041-010-0016-z 20505251

[25] Risso-Pascotto C, Pagliarini MS, Do Valle CB. A new basic chromosome number for the genus *Brachiaria* (Trin.) Griseb. (Poaceae: Panicoideae: Paniceae). Genet Resour Crop Evol. 2006;53:7–10.

[26] Vigna BBZ, Santos JCS, Jungmann L, do Valle CB, Mollinari M, Pastina MM, et al. Evidence of allopolyploidy in *Urochloa humidicola* based on cytological analysis and genetic linkage mapping. PLoS One. 2016;11:e0153764. Available from: 10.1371/journal.pone.0153764. 10.1371/journal.pone.0153764 PMC4841517 27104622

[27] Valle CB, Glienke C. New sexual accessions in *Brachiaria*. Apomixis News Let. 1991;3:11–3.

[28] Penteado MIDO, dos Santos ACM, Rodrigues IF, Valle CB, Seixas MAC, Esteves A. Determinacao de ploidia e avaliacao da quantidade de DNA total em diferentes especies do genero *Brachiaria*. EMBRAPA Gado de Corte: Campo Grande; 2000.

[29] Miles JW, Cardona C, Sotelo G (2006). Recurrent selection in a synthetic brachiariagrass population improves resistance to three spittlebug species. Crop Sci.

[30] Worthington ML, Miles JW. Reciprocal Full-sib Recurrent Selection and Tools for Accelerating Genetic Gain in Apomictic *Brachiaria*. In: Budak H, Spangenberg G, editors. Molecular Breeding of Forage and Turf. Cham: Springer; 2015. p. 19–30.

[31] Pedraza Garcia FP. Hacia la localización del gen de apomixis en *Brachiaria* usando marcadores moleculares RAPD [thesis]. Palmira: Universidad Nacional de Colombia; 1995.

[32] Zorzatto C, Chiari L, Araújo Bitencourt G, Valle CB, Campos Leguizamón GO, Schuster I, et al. Identification of a molecular marker linked to apomixis in *Brachiaria humidicola* (Poaceae). Plant Breed. 2010;129:734–6.

[33] Jungmann L, Vigna BBZ, Boldrini KR, Sousa ACB, Valle CB, Resende RMS, et al. Genetic diversity and population structure analysis of the tropical pasture grass *Brachiaria humidicola* based on microsatellites, cytogenetics, morphological traits, and geographical origin. Genome. 2010;53:698–709. 10.1139/g10-055 20924419

[34] Young BA, Sherwood RT, Bashaw EC (1979). Cleared-pistil and thick-sectioning techniques for detecting aposporous apomixis in grasses. Can J Bot.

[35] Nakagawa H. Embryo sac analysis and crossing procedure for breeding apomictic guineagrass (*Panicum maximum* Jacq.). Japan Agric Res Q. 1990;24:163–8.

[36] Valle CB, Savidan YH, Jank L. Apomixis and sexuality in *Brachiaria decumbens* Stapf. Nice: Proceedings of the XVI International Grasslands Congress; 1989. p. 407–8.

[37] Shure M, Wessler S, Fedoroff N (1983). Molecular identification and isolation of the waxy locus in maize. Cell.

[38] Vos P, Hogers R, Bleeker M, Reijans M, van de Lee T (1995). A new technique for DNA fingerprinting. Nucleic Acids Res.

[39] Faircloth BC (2008). MSATCOMMANDER: detection of microsatellite repeat arrays and automated, locus-specific primer design. Mol Ecol Resour.

[40] Untergasser A, Nijveen H, Rao X, Bisseling T, Geurts R, Leunissen JAM (2007). Primer3Plus, an enhanced web interface to Primer3. Nucleic Acids Res.

[41] Schuelke M. An economic method for the fluorescent labeling of PCR fragments. Nat Biotech. 2000;18:233-4. 10.1038/72708 10657137

[42] Yamanaka N, Hasran M, Xu DH, Tsunematsu H, Idris S, Ban T. Genetic relationship and diversity of four *Mangifera* species revealed through AFLP analysis. Genet Resour Crop Evol. 2006;53:949–54.

[43] Wenzl P, Mancilla LI, Mayer JE, Albert R, Rao IM. Simulating infertile acid soils with nutrient solutions. Soil Sci Soc Am J. 2003;67:1457-69.

[44] Luo R, Liu B, Xie Y, Li Z, Huang W, Yuan J, et al. SOAPdenovo2: an empirically improved memory-efficient sort read de novo assembler. Gigascience. 2012;1:18. 10.1186/2047-217X-1-18 PMC3626529 23587118

[45] Langmead B, Salzberg SL (2012). Fast gapped-read alignment with bowtie 2. Nat Methods.

[46] Duitama J, Quintero JC, Cruz DF, Quintero C, Hubmann G, Foulquié-Moreno MR, et al. An integrated framework for discovery and genotyping of genomic variants from high-throughput sequencing experiments. Nucleic Acids Res. 2014;42:e44. 10.1093/nar/gkt1381 PMC3973327 24413664

[47] Heffelfinger C, Fragoso CA, Moreno MA, Overton JD, Mottinger JP, Zhao H (2014). Flexible and scalable genotyping-by-sequencing strategies for population studies. BMC Genomics.

[48] Lu F, Lipka AE, Glaubitz J, Elshire R, Cherney JH, Casler MD (2013). Switchgrass genomic diversity, ploidy, and evolution: novel insights from a network-based SNP discovery protocol. PLoS Genet.

[49] Li X, Wei Y, Acharya A, Jiang Q, Kang J, Brummer EC. A saturated genetic linkage map of autotetraploid alfalfa (*Medicago sativa* L.) developed using genotyping-by-sequencing is highly syntenous with the *Medicago truncatula* genome. G3. 2014;4:1971–9. 10.1534/g3.114.012245 PMC4199703 25147192

[50] Melo ATO, Bartaula R, Hale I (2016). GBS-SNP-CROP: a reference-optional pipeline for SNP discovery and plant germplasm characterization using variable length, paired-end genotyping-by-sequencing data. BMC Bioinformatics.

[51] Van Ooijen JW. Multipoint maximum likelihood mapping in a full-sib family of an outbreeding species. Genet Res. 2011;93:343–9. 10.1017/S0016672311000279 21878144

[52] Voorrips RE (2002). MapChart: software for the graphical presentation of linkage maps and QTLs. J Hered.

[53] Zhang G, Liu X, Quan Z, Cheng S, Xu X, Pan S, et al. Genome sequence of foxtail millet (*Setaria italica*) provides insights into grass evolution and biofuel potential. Nat Biotechnol. 2012;30:549–54. 10.1038/nbt.2195 22580950

[54] Mason AS, Batley J, Bayer PE, Hayward A, Cowling WA, Nelson MN. High-resolution molecular karyotyping uncovers pairing between ancestrally related *Brassica* chromosomes. New Phytol. 2014;202:964–74. 10.1111/nph.12706 24471809

[55] Kriegner A, Cervantes JC, Burg K, Mwanga ROM, Zhang DP (2003). A genetic linkage map of sweetpotato *Ipomoea batatas* (L.) Lam. based on AFLP markers. Mol Breed.

[56] Hackett CA, Milne I, Bradshaw JE, Luo Z (2007). TetraploidMap for windows: linkage map construction and QTL mapping in autotetraploid species. J Hered.

[57] Risso-Pascotto C, Pagliarini MS, do Valle CB. Microsporogenesis in *Brachiaria dictyoneura* (Fig. & De not.) Stapf (Poaceae: Paniceae). Genet Mol Res. 2006;5:837–45. 17183491

[58] Boldrini KR, Adamowski EV, Silva N, Pagliarini MS, Valle CB. Meiotic behavior in nonaploid accessions of *Brachiaria humidicola* (Poaceae) and implications for breeding. Genet Mol Res. 2011;10:169–76. 10.4238/vol10-1gmr990 21308658

[59] Bernini C, Marin-Morales MA. Karyotype analysis in *Brachiaria* (Poaceae) species. Cytobios. 2001;104:157–71. 11318511

[60] Stanford EH, Clement WM. Cytology and crossing behavior of a haploid alfalfa plant. Agron J. 1958;50:598-92.

[61] Boldrini KR, de Adamowski EV, Message H, Calisto V, Pagliarini MS, Valle CB. Meiotic behavior as a selection tool in the breeding of *Brachiaria humidicola* (Poaceae). Euphytica. 2011;182:317–24.

[62] Chester M, Gallagher JP, Symonds VV, Cruz da Silva AV, Mavrodiev EV, Leitch AR (2012). Extensive chromosomal variation in a recently formed natural allopolyploid species, *Tragopogon miscellus* (Asteraceae). Proc Natl Acad Sci.

[63] Xiong Z, Gaeta RT, Pires JC (2011). Homoeologous shuffling and chromosome compensation maintain genome balance in resynthesized allopolyploid *Brassica napus*. Proc Natl Acad Sci.

[64] Mestiri I, Chagué V, Tanguy A, Huneau C, Huteau V, Belcram H, et al. Newly synthesized wheat allohexaploids display progenitor-dependent meiotic stability and aneuploidy but structural genomic additivity. New Phytol. 2010;186:86–101. 10.1111/j.1469-8137.2010.03186.x 20149116

[65] Hopkinson J, Souza F, Diulgheroff S, Ortiz A, Sanchez M. Reproductive physiology, seed production, and seed quality of *Brachiaria*. In: Miles JW, Maass BL, Valle CB, editors. *Brachiaria*: Biology, Agronomy, and Improvement. Palmira: CIAT; 1996. p. 125–40.

[66] Jank L, Barrios SC, Valle CB, Simeao RM, Alves GF (2014). The value of improved pastures to Brazilian beef production. Crop Pasture Sci.

[67] Souza VF, Pagliarini MS, Valle CB, Bione NCP, Menon MU. Meiotic behavior of *Brachiaria decumbens* hybrids. Genet Mol Res. 2015;14:12855–65. 10.4238/2015.October.21.5 26505437

[68] Pessino SC, Evans C, Ortiz JPA, Armstead I, Valle CB, Hayward MD. A genetic map of the apospory-region in *Brachiaria* hybrids: Identification of two markers closely associated with the trait. Hereditas. 1998;128:153–8.

[69] Pessino SC, Ortiz JPA, Leblanc O, Valle CB, Evans C, Hayward MD. Identification of a maize linkage group related to apomixis in *Brachiaria*. Theor Appl Genet. 1997;94:439–44.

[70] Sapkota S, Conner JA, Hanna WW, Simon B, Fengler K, Deschamps S, et al. In silico and fluorescence in situ hybridization mapping revealscollinearity between the *Pennisetum squamulatum* apomixis carrier-chromosome and chromosome 2 of sorghum and foxtail millet. PLoS One. 2016;11:e0152411. 10.1371/journal.pone.0152411 PMC4816547 27031857

[71] Jessup RW, Burson BL, Burow GB, Wang YW, Chang C, Lia Z (2002). Disomic inheritance, suppressed recombination, and allelic interactions govern apospory in buffelgrass as revealed by genome mapping. Crop Sci.

[72] Ozias-Akins P, Akiyama Y, Hanna WW. Molecular characterization of the genomic region linked with apomixis in *Pennisetum*/*Cenchrus*. Funct Integr Genomics. 2003;3:94–104. 10.1007/s10142-003-0084-8 12827522

